# Role of quantitative imaging biomarkers in an early FDG-PET/CT for detection of immune-related adverse events in melanoma patients: a prospective study

**DOI:** 10.2478/raon-2024-0045

**Published:** 2024-09-15

**Authors:** Nezka Hribernik, Katja Strasek, Daniel T Huff, Andrej Studen, Katarina Zevnik, Katja Skalic, Robert Jeraj, Martina Rebersek

**Affiliations:** Department of Medical Oncology, Institute of Oncology Ljubljana, Ljubljana, Slovenia; Faculty of Medicine, University of Ljubljana, Ljubljana, Slovenia; Faculty of Mathematics and Physics, University of Ljubljana, Ljubljana, Slovenia; University of Wisconsin Carbone Cancer Centre, Madison, WI, USA; Department of Medical Physics, University of Wisconsin-Madison, Madison, WI, USA; Experimental Particle Physics Department, Jožef Stefan Institute, Ljubljana, Slovenia; Department of Nuclear Medicine, Institute of Oncology Ljubljana, Ljubljana, Slovenia

**Keywords:** ^18^F-FDG PET/CT, immune-checkpoint inhibitors, immune-related adverse effects, quantitative imaging biomarkers, SUV percentiles

## Abstract

**Background:**

To evaluate the role of the novel quantitative imaging biomarker (QIB) SUV_X%_ of ^18^F-FDG uptake extracted from early ^18^F-FDG-PET/CT scan at 4 weeks for the detection of immune-related adverse events (rAE) in a cohort of patients with metastatic melanoma (mM) patients receiving immune-checkpoint inhibitors (ICI).

**Patients and methods:**

In this prospective non-interventional, one-centre clinical study, patients with mM, receiving ICI treatment, were regularly followed by ^18^F-FDG PET/CT. Patients were scanned at baseline, early point at week four (W4), week sixteen (W16) and week thirty-two (W32) after ICI initiation. A convolutional neural network (CNN) was used to segment three organs: lung, bowel, thyroid. QIB of irAE - SUV_X%_ - was analyzed within the target organs and correlated with the clinical irAE status. Area under the receiver-operating characteristic curve (AUROC) was used to quantify irAE detection performance.

**Results:**

A total of 242 ^18^F-FDG PET/CT images of 71 mM patients were prospectively collected and analysed. The early W4 scan showed improved detection only for the thyroid gland compared to W32 scan (p=0.047). The AUROC for detection of irAE in the three target organs was highest when SUV_X%_ was extracted from W16 scan and was 0.76 for lung, 0.53 for bowel and 0.81 for thyroid. SUV_X%_ extracted from W4 scan did not improve detection of irAE compared to W16 scan (lung: p = 0.54, bowel: p = 0.75, thyroid: p = 0.3, DeLong test), as well as compared to W32 scan in lungs (p = 0.32) and bowel (p = 0.3).

**Conclusions:**

Early time point ^18^F-FDG PET/CT at W4 did not lead to statistically significant earlier detection of irAE. However, organ ^18^F-FDG uptake as quantified by SUV_X%_ proved to be a consistent QIB of irAE. To better assess the role of ^18^F-FDG PET/CT in irAE detection, the time evolution of ^18^F-FDG PET/CT quantifiable inflammation would be of essence, only achievable in multi centric studies.

## Introduction

Immunotherapy with immune checkpoint inhibitors (ICI) is considered the standard of care for the treatment of unresectable stage III and IV metastatic melanoma (mM). It leads to high response rates and improvement of survival in this group of cancer patients.^1,2,3,4^ ICI treatments can cause different immune-related adverse events (irAE), where an immune response is generated against healthy tissue.^5,6,7^ Nearly one quarter of ICI treated patients experience higher grades of irAE that require hospitalization, 26% due to immune-related pneumonitis (irPneumonitis) and 17% due to immune-related colitis (irColitis).^8^ Consequently, prolonged immune suppressive treatment is needed, which can significantly endanger patient health and reduce the efficacy of ICI treatment.

The immense progress of ICI has brought different challenges, one of them being assessment of treatment efficacy and management of variety of irAE. Positron emission tomography/computed tomography with [^18^F]fluoro-2-deoxy-D-glucose (^18^F-FDG PET/CT) is a non-invasive method, commonly used in diagnosis, staging, and treatment response monitoring in mM. Its role in the treatment process is being recognized through different guidelines and emerging evidence.^9,10^

In addition to monitoring response, ^18^F-FDG PET/CT can detect certain irAE, some even before they clinically or biochemically manifest.^11,12,13^ Early detection or even prediction of irAE is of great interest, as this could contribute to better treatment.^11,14^ To date, no study has been published investigating whether a systematic search for irAE with ^18^F-FDG PET/CT imaging as early as four weeks after the start of ICI treatment would help in early detection of irAE. However, several studies investigated utility of different ^18^F-FDG PET/CT timing. A small prospective study with 20 mM patients evaluated if ^18^F-FDG PET/CT at days 21–28 would help predicting response to ICI in mM patients. It showed that increased ^18^F-FDG uptake early during ICI treatment may be associated with immune activation and favourable outcome.^15^ No analysis of irAE was reported. A similar study investigated prognostic value of ^18^F-FDG PET/CT after two cycles of ICI administration.^16^ They looked also for patterns of ^18^F-FDG uptake suggestive of irAE and found that the emergence of PET signs of irAE was not associated with patient survival. Another recent study analysed the usefulness of ^18^F-FDG PET/CT performed even earlier, one week after starting the treatment with pembrolizumab of mM patients. The study was aimed to evaluate metabolic changes in melanoma metastases one week after treatment start in order to predict the response to treatment. No evaluation of organs for irAE was reported.^17^

The segmentation of metastatic lesions and organs on ^18^F-FDG PET/CT with deep learning-based convolutional neural network (CNN) has recently been developed.^18,19^ In a previous work by our group of 58 mM patients receiving ICI, a novel quantitative imaging biomarker (QIB) of irAE development was proposed – percentiles of SUV distribution (SUV_X%_) of ^18^F-FDG uptake within the target organs.^11^ Three target organs were considered for identification of irAE: lung, bowel and thyroid. These target organs were segmented using a CNN, and SUV_X%_ of ^18^F-FDG uptake within the target organs were correlated with the clinical irAE status. It was shown that increased ^18^F-FDG uptake within irAE-affected organs provides predictive information about the development of irAE and represents a potential QIB for irAE. Some irAE were detected on ^18^F-FDG PET/CT before clinical symptoms appeared.^11^

The aim of this prospective study was to evaluate the role of the QIB SUV_X%_ of ^18^F-FDG uptake extracted from early ^18^F-FDG PET/CT scans for the detection of irAE in a cohort of patients with mM, treated with ICI. ^18^F-FDG PET/CT imaging was performed at an early timepoint at week 4 (W4) and two consecutive scans at week 16 (W16) and week 32 (W23) after ICI initiation. We hypothesized that quantitative analysis of three organs of interest (lung, bowel and thyroid) on week 4 ^18^F-FDG PET/CT would give information about irAE detection that precede clinical diagnosis and outperform later scans.

## Patients and methods

### Patient population and study protocol

We have been conducting a noninterventional, prospective study with primary endpoint of quantitative analysis of ^18^F-FDG PET/CT scans in metastatic melanoma patients for detection of irAE. The patients were treated per standard of care with ICI with anti-cytotoxic T-lymphocyte-associated antigen 4 (anti-CTLA-4) and/or anti programmed death-1 (anti-PD-1) treatment in first and second line of systemic treatment at the Institute of Oncology Ljubljana (OIL), Slovenia. Eligible patients were older than 18 years and had a stage III.D unresectable melanoma or a stage IV melanoma. All patients had an Eastern Cooperative Oncology Group (ECOG) performance status score of 0 or 1. Patients were eligible if they had the baseline ^18^F-FDG PET/CT performed within four weeks prior to ICI treatment initiation. Key exclusion criteria included symptomatic brain metastases and malignant diseases other than melanoma. Written consent was received from all participants.

Patients were monitored with regular ^18^F-FDG PET/CT. A baseline scan was performed within four week prior toICI initiation. After treatment initiation, ^18^F-FDG PET/CT was performed at W4 (+/− 5 days), W16 (+/− 7 days), W32 (+/− 7 days) and every 16 weeks after. Because the W4 ^18^F-FDG PET/CT was performed for investigational purposes, the scan data was not necessarily used to guide treatment decisions. In case of clear progression such as hyperprogression^20^, systemic treatment was allowed to be switched based on the decision of the medical oncologists leading the treatment. Clinical data and images included in this analysis were data obtained from disease diagnosis up to the W32 scan plus an additional 4 weeks, allowing for clinical detection of up to W32 clinically unobserved irAE in monitored organs, or 252 day after treatment start in cases where the patient did not have a W32 scan.

All ^18^F-FDG PET/CT data acquired before and during ICI treatment and all clinical data was collected for review. Grading of irAE was assigned prospectively following Common Terminology Criteria for Adverse Events (CTCAE, v.5.0).^21^ Imaging and clinical data were stored in a secure LabKey database server.^22^

The clinical protocol was approved by the Ethics Committee ERIDEK-0034/2020 and the Clinical Trials Protocol Review Committee ERIDKSOPKR-0032/2020 at the Institute of Oncology Ljubljana, and by Commission of the Republic of Slovenia for Medical Ethics (approval number: 0120-256/2020-14, September 15^th^ 2020). It was conducted following the ethical standards defined by the Declaration of Helsinki and the International Conference on Harmonisation Guidelines for Good Clinical Practice. The study was registered with the ClinicalTrials.gov under the registration number NCT06207747.

The study was conducted with acknowledgement and consent of the subjects. All patients have signed informed consent for treatment and consent allowing the usage of their data for scientific purposes. For publication of images in Figure 6 and 7, research participants provided informed consent.

### ^18^F-FDG PET/CT image acquisition

^18^F-FDG PET/CT scans were primarily performed for immunotherapy treatment response evaluation in melanoma patients. All ^18^F-FDG PET/CT scans were obtained on Biograph mCT PET/CT (Siemens, Knoxville, TN); one scan was obtained at University Clinical Centre Maribor (UKC MB), two at University Clinical Centre Ljubljana (UKC LJ) and the rest were acquired at the Institute of Oncology Ljubljana (OIL) following institutional protocols. Imaging protocol required patients to fast for 6 hours prior to injection of the radiotracer and have a blood glucose level below 10 mmol/L at the time of the scan. Patients were required to hold all diabetic medication, including metformin, for 6 hours prior to radiotracer injection. All scans were acquired per Standard of Care (SOC). CT that meets RECIST analysis needs was acquired according to adjusted protocol including SAFIR reconstruction to minimize dose. Following reconstruction, PET images were normalized by patient weight and injected dose to compute Standardized Uptake Values (SUV). More information on scanning parameters for each scanner can be found in Supplementary Table 1.

### ^18^F-FDG PET/CT image analysis

Before image analysis, images were resampled to a cubic 2-mm grid (2mm × 2mm × 2mm) using linear resampling and normalized to have a mean of 0 and variance of 1 within patient to match DeepMedic model developed for Hribernik *et al*.^11^

To quantify organ uptake, a 3D CNN was used to segment three target organs: lung, bowel and thyroid. The architecture used was nnUNet.^23^ The network was trained to perform inference on CT component from the ^18^F-FDG-PET/CT scans to produce contours of all three organs and was trained on same data as DeepMedic network, used in our previous pilot study.^11^

The organ contours, produced by nnUNet were applied to PET component of the ^18^F-FDG-PET/CT image to quantify ^18^F-FDG uptake in organs of interest. Percentiles X% of the organ SUV distribution (SUV_X%_) were extracted, as they were found to be potential biomarkers of irAE in retrospective study of mM patients and were found to be more reliable than SUV_max_.^11,14,24^ To quantify organ up-take, only the percentile, that was found to be optimal irAE biomarker in our pilot study in each organ was used: SUV_95%_ for lung and bowel, SUV_75%_ for thyroid.^11^

### Statistical analysis

Patients were divided into two groups for each analysed target organ (lung, bowel, thyroid): patients, who experienced irAE from time on ICI start up until the cut-off and patients who did not experience irAE during this period. All timing was calculated in days relative to start of treatment with ICI. In the group of patients experiencing irAE, statistics for all irAE events were calculated per type of irAE and per grade (any grade and high grade: grade 3–5 per CTCAE, v.5.0).^21^

From all clinically detected irAE observed in our follow-up period, the following were included in the analysis: immune-related pneumonitis (irPneumonitis), immune-related colitis (irColitis) and immune-related t hyroiditis (irThyroiditis). Receiver Operating Characteristic (ROC) analysis was performed to determine the ability of extracted SUV_X%_ in each organ to detect observed irAE on scans at W4, W16 and W32 individually. This was done by comparing the SUV_X%_ extracted from each timepoint scan with the clinical irAE status of the patient up until cut-off for all three analysed organs. Additionally, the maximum organ SUV_X%_ value, extracted from all available images (including baseline) up until W32, was investigated as a predictor of irAE. This ROC analysis was compared to ROC curves previously obtained in^11^ using the same methodology. Area under the ROC curve (AUROC) was calculated. The performance of different models was compared using DeLong test.

In the following part of the analysis, the threshold to differentiate between patients without irAE and those experiencing irAE based on SUV_X%_ and the normal SUV_X%_ range from analysis in pilot study were used.^11^ In that work, normal ranges of SUV_X%_ were for lung [0.7, 1.5] g/mL, bowel [1.8, 2.9] g/mL, and thyroid [0.9, 2.0] g/mL. Optimal thresholds used were: SUV_95%_ = 1.7 g/mL in lung, SUV_95%_ = 2.7 g/mL in bowel and SUV_75%_ = 2.1 g/mL in thyroid.

Target organ ^18^F-FDG uptake was assessed in those patients without clinically diagnosed irAE which had SUV_X%_ above threshold at any imaging timepoint. Medical oncologist and nuclear medicine specialist identified causes of higher uptake of ^18^F-FDG in the organ of interest in these patients using patient chart review. SUV_X%_ values in each organ of interest were compared between causes using Mann-Whitney U test.

Statistical comparison of irAE detection using SUV_X%_ and clinical detection was done. As a measure of instance when irAE was detected by ^18^F-FDG-PET/CT, we identified the first scan on which the SUV_X%_ in the organ of interest was above the mentioned threshold. We compared this instance to the timing of clinical diagnosis of irAE using Wilcoxon signed-rank test.

Image analysis and statistical testing was done using Python programming language version 3.7.16 (Python Software Foundation, https://www.python.org/). Statistically significant differences were observed when p value was below 0.05.

## Results

### Patient characteristics

Prospective study started with patient inclusion in September 2020. While the study is still ongoing, patient inclusion stopped in September 2022. The inclusion period of the study was initially planned to last 18 months but was extended for 6 months due to the COVID-19 pandemic. Altogether, 71 patients were included in the study; patient demographics are summarised in Table 1.

**TABLE 1.** Patient characteristics

	**Characteristics**	**Count (proportion %)**
**Number of patients**	Total	71 (100)
**Age; mean (+/−sd) (yr)**		62 (12)
**Gender**	Male	43 (61)
	Female	28 (39)
**ECOG performance status**	0	30 (42)
	1	41 (58)
**AJCC**	III.D	1 (1)
	M1a	16 (23)
	M1b	10 (14)
	M1c	32 (45)
	M1d	12 (17)
**Anatomic site of primary**	Cutaneous	58 (82)
	Ocular	4 (6)
	Mucosal	3 (4)
	Unknown primary	6 (8)
**Prior radical treatment**	Surgical resection	38 (54)
	Surgical resection + adjuvant RT	7 (10)
	Surgical resection + adjuvant RT + adjuvant ICI	4 (6)
	Surgical resection + adjuvant RT + adjuvant TKI	2 (3)
	Surgical resection + adjuvant TKI	2 (3)
	Surgical resection + adjuvant interferon alpha	2 (3)
	None	16 (23)
**Line of systemic treatment for metastatic disease**	1^st^ line	63 (89)
	2^nd^ line	8 (11)
**Baseline LDH**	Elevated	22 (31)
	Normal	49 (69)
**Actionable mutation**	*BRAF* wild type	21 (30)
	*BRAF* V600E	28 (39)
	*BRAF* V600K	10 (14)
	*BRAF* V600 - others	1 (1)
	*NRAS*	11 (16)
**Type of systemic treatment**	PD-1 inhibitors	47 (66)
	Combination of PD-1 and CTLA-4 inhibitors	24 (34)

AJCC = American Joint Classification of Cancer; BRAF = V-Raf Murine Sarcoma viral oncogene homolog B; CTLA-4 = Cytotoxic T-lymphocyte-associated antigen 4; ECOG = Eastern Cooperative Oncology Group; ICI = Immune checkpoint inhibitors; No = number of patients; NRAS = Neuroblastoma RAS viral homolog; PD-1 = Programmed death-1; SD = standard deviation; RT = radiotherapy; TKI = Tyrosine kinase inhibitors

### Clinical detection of irAE

Dates and grades of clinically detected irPneumonitis, irColitis and irThyroiditis were systematically collected. Number of events for all three types of irAE and median time to first clinical diagnosis of all irAE during observational period are given in Table 2.

**TABLE 2.** Clinical diagnosis of immune-related adverse events

**irAE**	**Any Grade No (%)**	**Grade 3–5 No (%)**	**Time to onset of irAE (mean ± SD) [days]**
**irPneumonitis**	3 (4)	0	104 ± 58
**irColitis**	4 (6)	2 (3)	101 ± 34
**Hypothyroidism**	10 (14)	0	91 ± 40
**Hyperthyroidism**	7 (10)	0	51 ± 50

irAE = immune-related adverse events; No = number of patients; SD = standard deviation

During our observational period, 10 patients (14%) developed hypothyroidism and 7 (10%) hyperthyroidism. We identified 13 patients (18%) to have developed irThyroiditis based on patterns of thyroid dysfunction.^25^

### ^18^F-FDG PET/CT

A total of 242 ^18^F-FDG PET/CT scans were collected and analyzed. Table 3 shows the number of scans per scan timepoint and timing of the scans. Out of 71 patients, 3 patients had only baseline scan and no further scans due to rapid disease progression and death prior to next imaging appointment. 13 patients had only two scans (baseline and W4); 5 due to disease progression and deterioration of their performance status, 8 due to disease progression and death prior to the next imaging appointment. 7 patients had only three scans (baseline, W4 and W16) due to disease progression and death prior to next imaging appointment. 48 patients had all scans, including W32.

**TABLE 3.** Timing of ^18^F-FDG PET/CT relative to immune-checkpoint inhibitors (ICI) treatment initiation

	**Number of patients**	**Range (min–max) (days)**	**Mean (days)**	**Median (days)**	**SD (days)**
**Pre-Treatment**	71	[−44, −1]	−16	−16	10
**W4 (28 days)**	68	[21, 62]	34	34	7
**W16 (112 days)**	55	[105,150]	115	112	8
**W32 (224 days)**	48	[196,280]	226	224	12

SD = standard deviation; W = week

### Detection of irAE from ^18^F-FDG PET/CT scan

To predict irAE status from maximum value of SUV_X%_ at any of the time points observed, the area under the receiver-operating characteristic curve (AUROC) was 0.57 in bowel, 0.7 in lung, 0.77 in thyroid. Compared to our previous analysis^11^, the performance of the model did not change (DeLong test, p > 0.05) in all three organs (AUROC: 0.79 in bowel, 0.98 in lung and 0.88 in thyroid in^11^).

**FIGURE 1.**
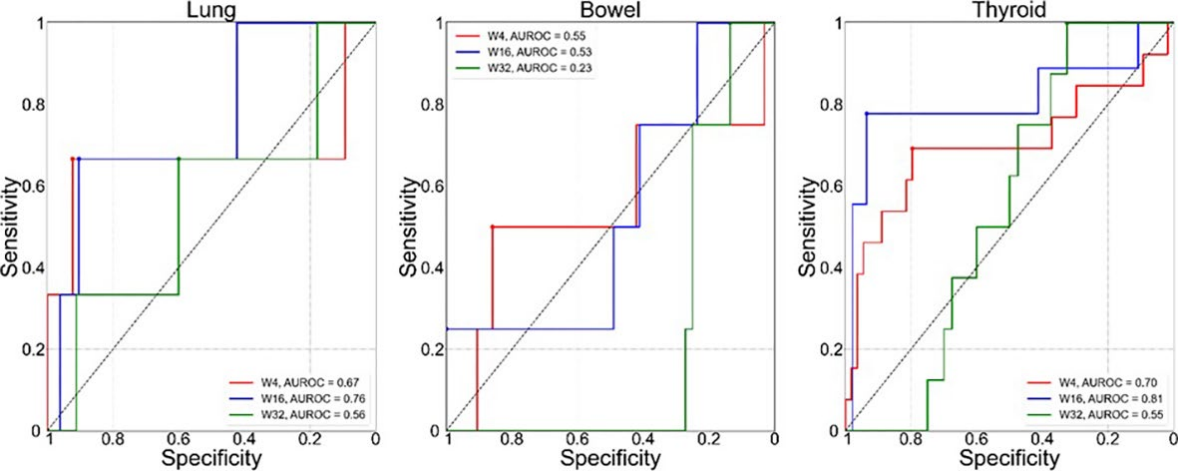
ROC curves for the optimal SUV percentile (SUV_X%_) for predicting irAE status in the three target organs:lung (left), bowel (middle), thyroid (right). Each plot shows comparison of ROC based on the value of SUV_X%_ extracted from W4 (red), W16 (blue) and W32 (green) ^18^F-FDG PET/CT. Values of corresponding area under the ROC curve (AUROC) are shown in the legends.

To evaluate the role of SUV_%_ extracted from early ^18^F-FDG PET/CT scan, the AUROC analysis of all time point scans was done. The AUROC for detection of adverse events in the three target organs was highest when SUV_X%_ was extracted from W16 scan and was 0.76 for lung, 0.53 for bowel and 0.81 for thyroid. SUV_X%_ extracted from W4 scan did not improve detection of irAE compared to W16 scan (lung: p = 0.54, bowel: p = 0.75, thyroid: p = 0.3, DeLong test), as well as compared to W32 scan in lungs (p = 0.32) and bowel (p = 0.3). For thyroid gland, the W4 scan marginally improved detection compared to W32 scan (p = 0.047). Comparison of detection using W16 and W32 scan in thyroid showed, that W16 was more optimal for detection then W32 (p = 0.02), but this was not observed in lungs and bowel (p = 0.09). The ROC curves for selected SUV_%_ extracted from W4, W16 and W32 scan for lung, bowel and thyroid are shown in Figure 1.

### irAE detection by ^18^F-FDG PET/CT versus clinical detection

**FIGURE 2.**
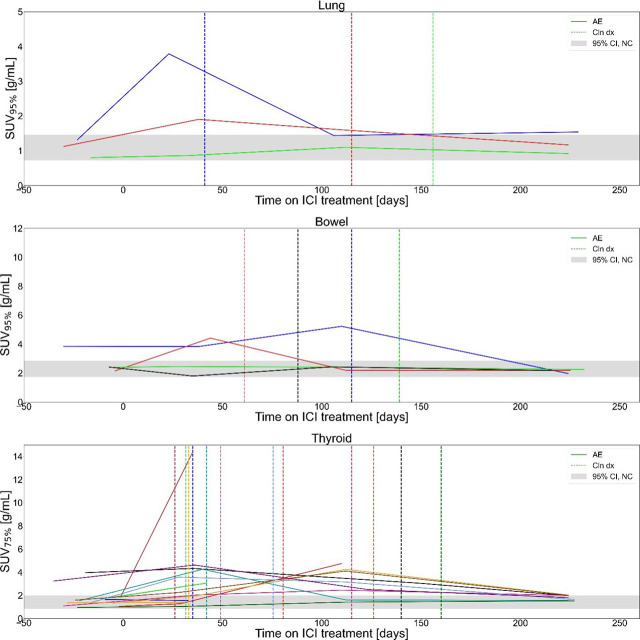
Longitudinal optimal SUV percentile (SUV_X%_) for patients with clinically detected immune-related adverse events (irAE) in bowel, lung and thyroid, included in study. The grey band indicates the 95% confidence interval for organ SUV_X%_ of patients who did not experience irAE in^11^. Colours were randomly selected for participants in each plot, vertical dashed lines of matching colour indicate dates of clinical irAE identification. ICI = Immune checkpoint inhibitors

**FIGURE 3.**
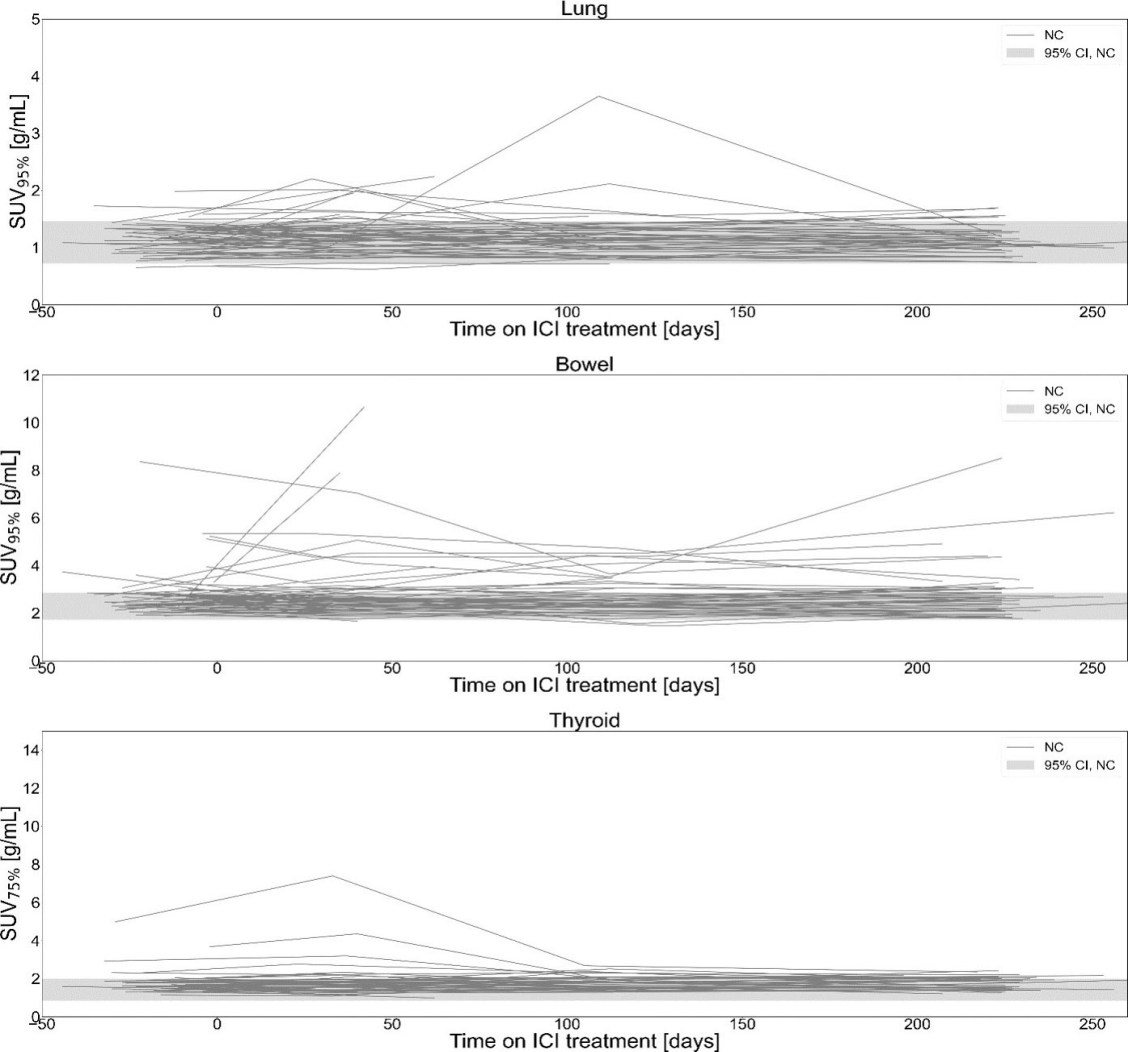
Longitudinal optimal SUV percentile (SUV_X%_) for patients without clinically detected immune-related adverse events (irAE) in bowel, lung and thyroid, included in study. The grey band indicates the 95% confidence interval for organ SUV_X%_ of patients who did not experience irAE in^11^. ICI = Immune checkpoint inhibitor

Longitudinal time series of SUV_X%_ for all patients included in the study are shown in Figure 2 and Figure 3. Patients, clinically diagnosed with irPneumonitis, irColitis and irThyroiditis, are shown with patient specific colours (each plot) in Figure 2; dates of clinical diagnosis of irAE for each participant are indicated with dashed vertical lines of the matching color. Longitudinal series of SUV_X%_ for patients that did not have clinically diagnosed irAE in any of the three organs are shown in Figure 3. The gray band indicated the 95% confidence interval for SUV_X%_ of patients who did not experience irAE in^11^.

**FIGURE 4.**
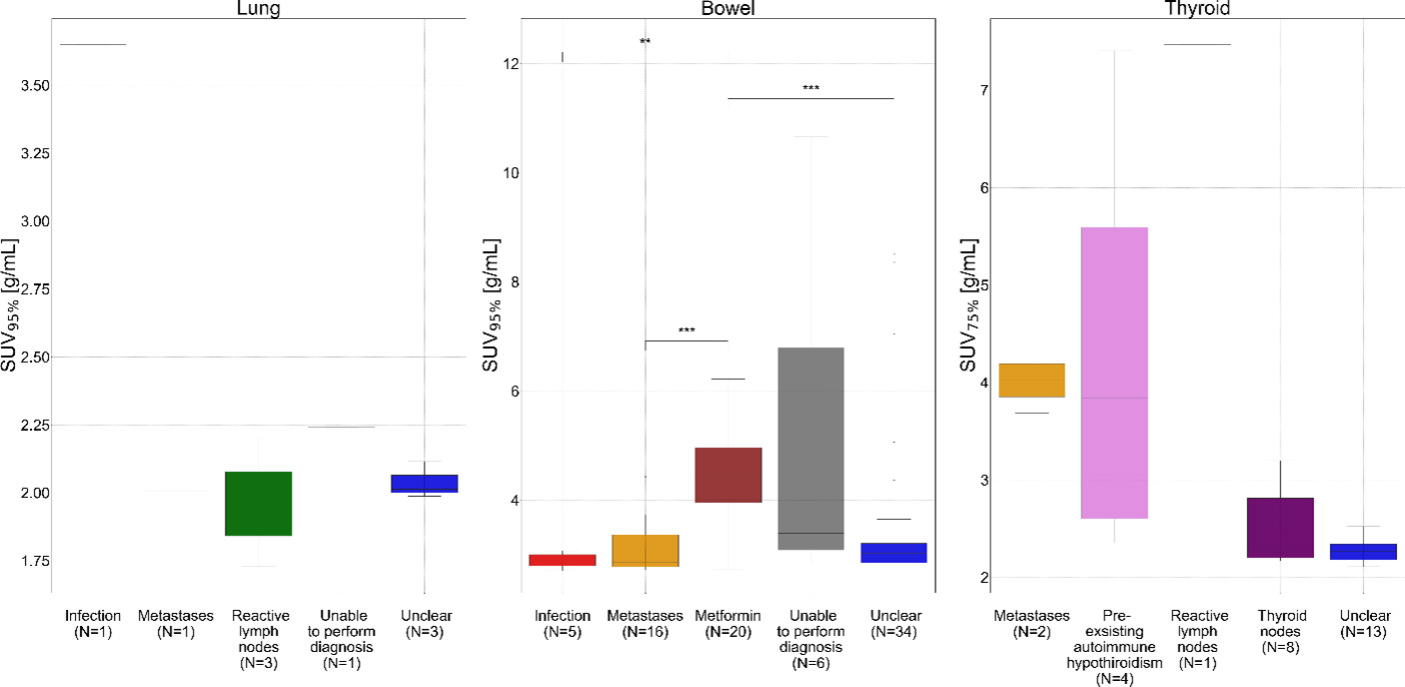
Boxplot shows values of optimal SUV percentile (SUV_X%_) above thresholds for immune-related adverse events (irAE) diagnosis in patients without clinically diagnosed irAE in lung, bowel, and thyroid. Values of SUV_X%_ are separated based on reason for SUV_X%_ above threshold, identified from patient’s chart. Each image timepoint and SUV_X%_ extracted from that image is considered as separate instance so multiple points can be contributed by a single patient. p-value was calculated using Mann-Whiteney U test. P-value is shown only for pairings with statistically significant differences * = shows p-value between 0.05 and 0.01; ** = for p-value between 0.01 and 0.001; *** = for p-value between 0.001 and 0.00001

We identified false positives cases where SUV_X%_ was above threshold without clinically confirmed irAE in any of the three organs. Values of SUV_X%_ above threshold, separated by reason, identified from patient’s chart, in lung, bowel and thyroid are shown in boxplot in Figure 4. Of 9 excursions in lung, 3 (33%) remained unclear, 3 (33%) were attributed to reactive lymph nodes and 1 (11%) to infection, metastases in that area, and inability to assess causes of high uptake each. In bowel, 34 excursions of 81 (42%) remained unclear, 20 (25%) were attributed to metformin and 16 (20%), 6 (7%), 5 (6%) to metastases, inability to assess causes of high up-take and infection, respectively. In thyroid, 13 of 28 (46%) excursions were unclear, 8 (29%), 4 (14%), 2 (7%) and 1 (4%) were attributed to thyroid nodes, pre-existing autoimmune thyroiditis, metastases in that area and reactive lymph nodes respectively. Inability to assess causes for SUV_X%_ above threshold was attributed to cases where poor patient’s condition and death prevented clinicians from assessing and confirming irAE. Cases where the cause could not be clarified by analysing patient’s charts were assigned to the group unclear.

Statistical analysis showed that patients on metformin had statistically significant higher SUV_95%_ in bowel compared to those identified with infection or metastases in bowel and unclear reason for higher uptake (Mann-Whitney-U test, p < 0.05; Figure 4). There were no statistically significant differences between other reasons and in other organs.

On average, the irAE were identified on ^18^FFDG-PET/CT using SUV_X%_ 38 days prior to clinical diagnosis; 48 days (range: 18, 77) for irPneumonitis, 81 (range: 17, 145) days for irColitis and 27 days (range: −47, 159) for irThyroiditis. Statistical comparison of the timing of clinical irAE diagnosis and timing of first SUV_X%_ above the threshold for all three observed organs showed that there is no statistically significant difference (N = 14, p = 0.1, Wilcoxon signed-rank test).

**FIGURE 5.**
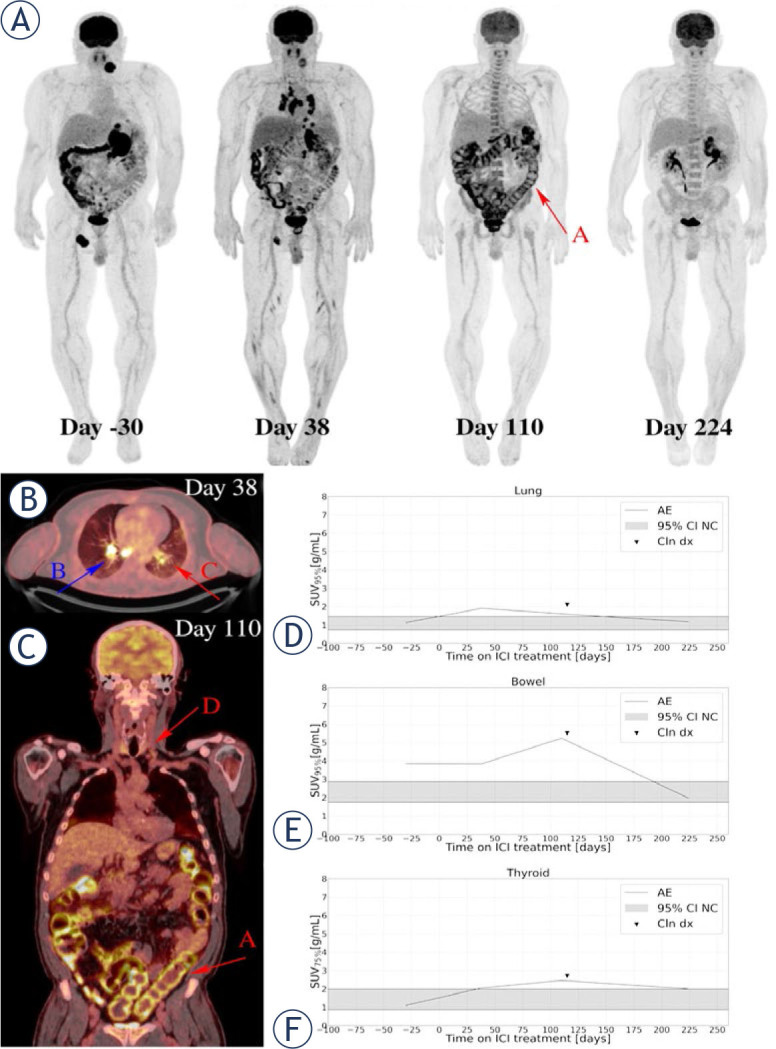
A 58-year-old patient, who was diagnosed in April 2022 with *NRAS* mutated melanoma of unknown primary was treated with ipilimumab/nivolumab combination. Serial ^18^F-FDG PET maximum intensity projections were obtained per study protocol **(A)**. The baseline image (day -30) showed sites of metastases in soft tissue, adrenal gland and spleen. Early time point (W4, day 38) image showed higher metabolic activity in mediastinal lymph nodes typical for granulomatous sarcoid like reaction, which is also seen on axial slices of the day 38 imaging study fused ^18^F-FDG PET/CT **(B, BLUE ARROW B)**, and diffuse lung opacities with mildly elevated ^18^F-FDG uptake **(B RED ARROW C)**. Patient reported of having no respiratory symptoms. Partial response in lymph nodes and in the spleen was described, and metabolic progression of the adrenal gland metastasis was observed. Day 110 (W16) image showed elevated ^18^F-FDG uptake in the thyroid gland **(C RED ARROW D)**, lung, and colon **(C, RED ARROW A)** reflecting multiple irAE. Coronal slices of the day 110 imaging study on fused ^18^F-FDG PET/CT **(C)**. On day 115 patient reported of having diarrhoea without respiratory or other symptoms, thyroid hormone laboratory test was pathological. Colonoscopy with biopsy was performed and examination by endocrinologist. IrColitis grade 2 and irThyroiditis grade 2 were confirmed. irPneumonitis grade 1 was confirmed based on imaging, no invasive procedure with bronchoscopy was performed for confirmation due to absence of respiratory symptoms and significant irColitis at the same time. After treatment with systemic corticosteroids, diarrhoea resolved. Images on day 224 (W32) were showing resolutions of all irAE and complete metabolic response in all metastatic lesions except progression in adrenal gland. This one metastatic lesion was surgical resected. Due to multiple irAE, the patient then stopped ICI treatment. Quantification of organ ^18^F-FDG PET uptake demonstrated elevated uptake in lung and preceded clinical symptoms **(D)**, elevated uptake in bowel corresponded with the time of clinical diagnosis of irColitis **(E)** as well as elevated uptake in thyroid corresponded with clinical diagnosis of irThyroiditis **(F)**. AE = adverse events(irAE

**FIGURE 6.**
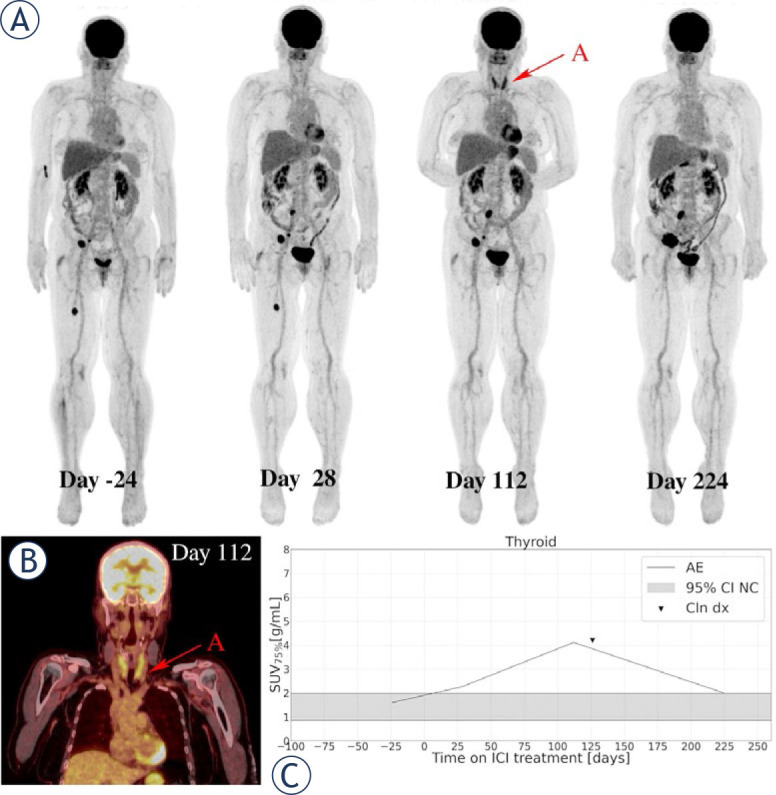
A 60-year-old female patient with metastatic *BRAF* V600E-mutated cutaneous melanoma, diagnosed in September 2021, was treated with pembrolizumab in the first-line setting. Serial ^18^F-FDG PET maximum intensity projections were obtained per protocol **(A)**. The baseline ^18^F-FDG PET/CT image (day -24) showed metastatic disease present in soft tissue. Day 28 (W4) imaging study showed moderately increased colon uptake and heterogeneous response of all metastatic lesions. Patient had no symptoms of irColitis. Image at day 112 (W16) showed again a heterogeneous response of melanoma metastases and marked increase in ^18^F-FDG uptake in the thyroid gland **(RED ARROW A)**, which can also be observed on coronal slice of the day 112 imaging study on fused ^18^F-FDG PET/CT **(B)**. Patient was confirmed to have irThyroiditis on day 126, based on thyroid hormone laboratory test and examination of endocrinologist. Day 224 (W32) image showed decrease in ^18^F-FDG uptake in the thyroid gland and a clear progression of melanoma metastases. Systemic treatment was changed to targeted treatment with BRAF and MEK inhibitor. Moderate increase in ^18^F-FDG PET uptake in the thyroid gland was seen already on W4 ^18^F-FDG PET, 98 days before clinical detected **(C)**. AE = adverse events(irAE

Images of two patients who experienced irP-neumonitis, irColitis, and irThyroiditis are highlighted in Figure 5 and Figure 6, respectively.

## Discussion

This is the first study that aimed to prospectively evaluate QIB SUV_X%_ on a W4 ^18^F-FDG PET/CT scan to detect irAE early during the systemic treatment with ICI. For now, there are only few published studies concerning the value of ^18^F-FDG PET/CT in detecting irAE, all performed retrospectively and none of them used ^18^F-FDG PET/CT performed at W4 for early evaluation.^26^ Recently published retrospective study determined the organ-specific accuracy of ^18^F-FDG PET/CT in detecting irAE in melanoma patients in adjuvant setting. They concluded that ^18^F-FDG PET/CT has a moderate to high sensitivity and specificity for diagnosis of irAE in lung, intestines and thyroid gland.^27^ Quantitative approach for the evaluation of ^18^F-FDG uptake in the target organs was till now used only in our retrospective analysis.^11^

We hypothesized that mM patients who were treated with ICI would demonstrate increased ^18^F-FDG uptake in the organs of interest before the time of clinical irAE diagnosis, which could be detectable as early as on W4 ^18^F-FDG PET/CT. We found that SUV_X%_ had no higher detection performance when extracted from W4 compared to W16 but had improved detection performance power when compared to W32. This might suggest that irAE are not yet visible or cannot be detected on W4 scan, but a later scan, possibly week 8, might be more appropriate for irAE detection. Interestingly enough, this is also the suggested time interval in guidelines for management of mM management.^27,28^

For mM patients receiving ICI, ^18^F-FDG PET/CT is primarily performed for disease response assessment. Data on optimum time of the first evaluation is scarce. In clinical practice it is usually performed after three to four cycles of ICI but can also be performed earlier of later during the course of the treatment, depending on the decision of medical oncologist and availability of ^18^F-FDG PET/CT scans.^28^ Of the three irAE investigated, the typical onset times and times to resolution of irThyroiditis fall exactly within W4 to W16, making it highly likely for irThyroiditis to be detected by either of the two early scans.^29^ For other two irAE, the onset of irAE can appear at any time during therapy, with a predominant period of 2–16 weeks from the commencement, depending on the type of ICI administered.^6,7,30^ It is hence unlikely to capture and detect irAE at its worse on routine ^18^F-FDG PET-CT images. Our data however shows that quantitative evidence of irAE can be seen in images as early as 80 days prior to clinical diagnosis of colitis. Due to rareness of occurrence in realistic study cohorts, multi-centre studies would be needed to describe the actual time-development curve of irAE. This could define the optimal timepoint of first on-treatment imaging for early diagnosis of irAE and earlier treatment intervention, which would in turn lower the possibility for detrimental impact of irAE on survival outcomes.^31^

In our study, there was no statistically significant difference in time of clinical diagnosis of irAE compared to its identification via SUV_X%_ extracted from ^18^F-FDG PET/CT. However, using SUV_X%_, irAE were on average identified as early as 81 days for irColitis, 48 days for irPneumonities and 27 days for irThyroditis prior to clinical diagnosis. In case of irThyroiditis, the small difference is primarily due to early clinical detection via regular laboratory control of thyroid hormones performed routinely before each ICI infusion. To a lesser extent, the short median time to onset of thyroid disfunction, usually less than two months.^29^ coincides well with W4 scans performed in our study. On the other hand, clinical detection of irColitis and irPneumonitis is driven by symptoms exhibited by the patient. Using SUV_X%_, irColitis and irPneumonitis could be first observed on ^18^F-FDG PET/CT while patient is still asymptomatic, which would enable closer monitoring and earlier treatment adjustment. This is illustrated in two cases of patients with SUV_X%_ in affected organs above threshold before clinical diagnosis of irAE in Figures 5 and 6.

The study also assessed SUV_X%_ for the detection of irAE, previously described in a pilot study from Hribernik *et al*. in a prospective setting.^11^ Comparing AUROC curves of SUV_X%_ for predicting irAE status in the three target organs showed similar predictive power to that first reported in our past publication (p > 0.05). This indicates that SUV_X%_ is a consistent QIB for irAE detection, as was previously concluded in Huff *et al*.^14^

SUV_X%_ is not a perfect QIB for identification of irAE, as it captures any SUV_X%_ above the threshold due to causes other than inflammation. Specifically, in the case of bowel, there are different patterns of physiological ^18^F-FDG uptake, and many different factors known and yet to be determined that possibly influence the physiological bowel ^18^F-FDG uptake, such as metformin and other antidiabetic therapy, gut microbiota, and bowel movement.^32,33,34^ Additionally, metastases present in organ of interest also impact ^18^F-FDG uptake and consequently, SUV_X%_. Although SUV_X%_ were hypothesized to be robust to metastases captured in the volume of interest, a more detailed studies, where segmented lesions would be excluded from organ uptake would help assess the impact of metastases uptake on SUV_X%_ robustness. Other factors that were found to impact value of SUV_X%_ and lead to false-positive results were also infections, reactive lymph nodes and overactive thyroid nodes before ICI treatment. Many of these possible false-positive cases were already described by Aide *et al*. in his review article.^12^

There are some limitations in this study. Variation in ^18^F-FDG PET/CT scan timing was present due to various reasons, mainly radiopharmaceutical outages, special interventions during the COVID-19 pandemic, and patient infections or deterioration of condition as the disease progressed. Also, not all patients were scanned on the same device, limiting quantification consistency across scanners. While potentially challenging, the inconsistencies were deemed acceptable due to comparable scanning protocols, low number of images performed elsewhere and similarity of scanners. For multicentric studies, scanner harmonization was shown to remove potential imaging bias. Another limitation is inconsistency in CNN for organ segmentation between previously published work and the present study. Our studies in consistency of AUROC are a strong indicator of SUV_X%_ robustness over segmentation methods.^35^

Our future aim is to use prospectively gathered data from this clinical study to analyse the potential role of a W4 ^18^F-FDG PET/CT for prediction of response to ICI. We will evaluate the prediction of response using lesion by lesion analysis, with the possible use of lesion segmentation with CNN and the analysis of different lesion based QIB.

## Conclusions

This is the first study to quantitatively analyse early timepoint ^18^F-FDG PET/CT using SUV_X%_ for detection of irAE in mM patients on ICI. While SUV_X%_ ware found to be a consistent biomarker of irAE, its use on a W4 ^18^F-FDG PET/CT had no higher performance compared to subsequent imaging. As the level of statistics exceeds a single-center studies, further larger studies are needed to investigate the use QIB of ^18^F-FDG PET/CT.

## Supplementary Material

Supplementary Material Details
