# Supplementary material for: Role of quantitative imaging biomarkers in an early FDG-PET/CT for detection of immune-related adverse events in melanoma patients: a prospective study
Source: Radiol Oncol. 2024 Sep 15;58(3):335–47. doi: 10.2478/raon-2024-0045 (PMC11406908; doi:10.2478/raon-2024-0045)
Supplement: Supplementary file 1 — Supplementary Material Details [file raon-2024-0045-sm.pdf]

sciendo
Radiology and Oncology | Ljubljana | Slovenia | www.radioloncol.com
RADIOLOGY AND ONCOLOGY

Role of quantitative imaging biomarkers in an early FDG-PET/CT for detection of immune-related adverse events in melanoma patients: a prospective study

Nezka Hribernik, Katja Strasek, Daniel T Huff, Andrej Studen, Katarina Zevnik, Katja Skalic, Robert Jeraj, Martina Rebersek

doi: 10.2478/raon-2024-0045

SUPPLEMENTALY TABLE 1. Scan parameters for PET/CTs of three scanners, used to obtain patient images at three different institutions

Institution		OIL	UKC Maribor	UKC Ljubljana
Scanner		Siemens Biograph mCT PET/CT	Siemens Biograph mCT	Siemens Biographs mCT
Number of scans		239	2	1
Protocol		5 MBq/kg (±10%)<br>Rest for 60 min<br>Scan from vertex to toes	4 MBq/kg (±10%)<br>Rest for 60 min<br>Scan from vertex to toes	3,5 MBq/kg (±10%)<br>Rest for 60 min<br>Scan from vertex to toes
CT	Number of slices	40	40	128
	Attenuation correction	Yes, anatomical localization	Yes, anatomical localization	Yes
	Tube current	120 kV	120 kV	N/A
	Tube voltage	80 mAs	70 mAs	N/A
	Dose modulation	Care dose 4D, Care kV	Care Dose 4D	N/A
	Collimation	16x1.2 mm	16x1.2 mm	N/A
	Pitch	1.2	0.8	N/A
	Reconstruction	3 mm slice thickness, 3 mm increment, abdominal window	3 mm slice thickness, 2 mm increment, abdominal window	3 mm slice thickness, 3 mm increment, abdominal window
	Reconstruction algorithm	SAFIRE	SAFIRE	N/A
	Reconstruction kernel	B40f	B30f	B30f
PET	Detector	LSO	LSO	N/A
	Data acquisition	2 min/bed position	2 min/bed position	N/A
	Image reconstruction algorithm	OSEM (2 iterations, 24 subsets), iterative TrueX (2 iterations, 21 subsets) which incorporates PSF correction and image matrix 200x200, TOF	Iterative TrueX (2 iterations, 21 subsets) which incorporates PSF and image matrix 200x200	OSEM (2 iterations, 21 subsets) with PSF and TOF, IMAGE MATRIX 400X400
	Number of rings	4	N/A	3

LSO = luthetium oxyorthoscilicate scintillator; N/A not available; OIL = Institute of Oncology Ljubljana; OSEM = ordered subset expectation maximization; PSF = point spread function; SAFIRE = sinogram affirmed iterative reconstruction; TOF = time-of-flight; UKC = University Clinical Center

The parameters for UKC Ljubljana were extracted from DICOM tags.
